# Traditional Fermented Beverages of Mexico: A Biocultural Unseen Foodscape

**DOI:** 10.3390/foods10102390

**Published:** 2021-10-09

**Authors:** César Ojeda-Linares, Gonzalo D. Álvarez-Ríos, Carmen Julia Figueredo-Urbina, Luis Alfredo Islas, Patricia Lappe-Oliveras, Gary Paul Nabhan, Ignacio Torres-García, Mariana Vallejo, Alejandro Casas

**Affiliations:** 1 Instituto de Investigaciones en Ecosistemas y Sustentabilidad, Universidad Nacional Autónoma de México, Antigua Carretera a Pátzcuaro 8701, Col. San José de la Huerta, Morelia 58190, Mexico; cojeda@cieco.unam.mx (C.O.-L.); galvarez@cieco.unam.mx (G.D.Á.-R.); lislas@cieco.unam.mx (L.A.I.); 2 Cátedras CONACYT-Laboratorio de Genética, Área Académica de Biología, Instituto de Ciencias Básicas e Ingeniería, Universidad Autónoma del Estado de Hidalgo, Mineral de la Reforma 42184, Mexico; figueredocj@gmail.com; 3 Instituto de Biología, Tercer Circuito Exterior, S/N Ciudad Universitaria, Coyoacán, Ciudad de México 04510, Mexico; lappe@ib.unam.mx; 4 The Southwest Center of University of Arizona, 1401 E. First St., Tucson, AZ 85721, USA; gpnabhan@arizona.edu; 5 Escuela Nacional de Estudios Superiores Unidad Morelia, Universidad Nacional Autónoma de México, Antigua Carretera a Pátzcuaro No. 8701, Col. Ex Hacienda de San José de la Huerta C.P., Morelia 58190, Mexico; itorresg@enesmorelia.unam.mx; 6 Jardín Botánico, Instituto de Biología, Universidad Nacional Autónoma de México, Tercer Circuito Exterior, S/N Ciudad Universitaria, Coyoacán, Ciudad de México 04510, Mexico

**Keywords:** ethnobiology, ethnozymology, fermentation, Mesoamerican biocultural heritage, traditional food systems

## Abstract

Mexico is one of the main regions of the world where the domestication of numerous edible plant species originated. Its cuisine is considered an Intangible Cultural Heritage of Humanity and ferments are important components but have been poorly studied. Traditional fermented foods are still diverse, but some are endangered, requiring actions to promote their preservation. Our study aimed to (1) systematize information on the diversity and cultural history of traditional Mexican fermented beverages (TMFB), (2) document their spatial distribution, and (3) identify the main research trends and topics needed for their conservation and recovery. We reviewed information and constructed a database with biocultural information about TMFB prepared and consumed in Mexico, and we analyzed the information through network approaches and mapped it. We identified 16 TMFB and 143 plant species involved in their production, species of Cactaceae, Asparagaceae, and Poaceae being the most common substrates. Microbiological research has been directed to the potential biotechnological applications of *Lactobacillus*, *Bacillus,* and *Saccharomyces*. We identified a major gap of research on uncommon beverages and poor attention on the cultural and technological aspects. TMFB are dynamic and heterogenous foodscapes that are valuable biocultural reservoirs. Policies should include their promotion for conservation. The main needs of research and policies are discussed.

## 1. Introduction

The consumption of ferments by humans probably started unintentionally since fermentation occurs in nature as a common process [1]. However, whatever its origin, the consumption of fermented foods has accompanied people since ancient times [2]. The obtaining of fermented products with desirable attributes and the conditions determining them were eventually recognized and deliberately selected and favored [3]. Together with roasting, fermentation is perhaps one of the oldest practices and techniques used by humans to process plant and animal materials to prepare food [4]. Fermented products commonly include foods, among them beverages, but dyes, fibers, and other products involving fermentation have also been recorded since remote past times [5,6,7]. At some point in the human cultural history, knowledge over the fermentation process increased, hygienic practices were developed, and new techniques were adopted, outstandingly, the use of inoculums; subsequently, the manufacturing of fermented foods and beverages became the responsibility of skilled craftspersons who were mainly responsible for developing and improving the technologies for making fermented foods. Those crafters have been recognized in the literature as traditional fermentation managers [7].

Currently, a small fraction of species of organisms are predominantly used in the human diet worldwide, and this fact makes the hegemonic food systems vulnerable [8]. Fermentation plays an important role in improve the shelf life of several products, as well as to increase the diversity of food supplies and their nutritional value [9]. Therefore, the goals of diversifying food systems of the world have a key support in ferments. Most traditional ferments represent keystone products in several countries because they are expressions of interrelationships between biological and cultural diversity, and dynamic biocultural knowledge changing according to adaptive processes [9,10].

Fermented products are outputs of complex interactions of three main components, substrates, microorganisms causing fermentation, and humans who drive the process and value the final products [11,12]. Humans have observed and directed the fermentation process by choosing substrates, and selecting specific attributes of their beverages looking for pleasant-tasting, nicely textured, and long-lasting products [7,12,13,14]. Through time, humans have improved their techniques by experimenting with local substrates, adding others, testing variations in the general conditions of the process, and evaluating the product resulting from that process [11,12,13,14,15,16]. These biocultural practices have generated thousands of food types, including beverages, throughout human history and it is a process that continues evolving to the present day.

The fermented foods and beverages were used long before any awareness of microbiology and biochemistry [17], but now these sciences provide research tools that make it possible to identify the crucial role of microorganism assemblages in fermentation, as well as the techniques to direct fermentation according to human purposes. Microorganisms are present in substrates (plant or animal) or could be added as consortiums of microorganisms known as starter cultures. These starters may simply be small batches of previous fermentations, stored and added to new substrates to guarantee a faster and homogeneous fermentation that yields specific sensorial attributes [18,19,20]. Moreover, these may be microorganisms that remain in the containers where previous fermentation processes occurred, and people leave them there for new fermentation [21]. Environmental conditions are variable during fermentation, which confers variable quality and sensorial characteristics to the final product. However, traditional producers perform different practices to reduce the effect of environmental heterogeneity during the fermentation processes [7,15,22,23], while commercially fermented products are highly technified and the outcome is standardized.

Fermented beverages are among the most iconic fermented foods worldwide; these are essential components of local diets in many cultures and are mainly prepared from plant substrates [24,25,26,27]. It has been reported that through fermentation, foods may be improved, preserved, and their organoleptic properties enhanced [11]. Fermented products can have an important sociocultural role [15,24,25,26,27], consumed during holidays, ceremonies, and rituals, and linked to fruiting seasons and the harvest of agricultural products [27]. Both consumers and producers decide if the quality of a fermented product is acceptable considering their attributes and determine if it is kept or not in their diet [11,12,13,14,25,26,27]. Despite the importance of fermented products around the world, efforts for tracking their loss and actions to recover their production are uncommon. However, there are iconic and inspiring cases such as that in northeastern Poland, where researchers registered signs of recovering the tradition of producing juniper beers [28].

The traditional ecological knowledge involved in the preparation of fermented products appears to be at risk because of the overreliance on the massive campaigns of commercially produced beverages, which reach rural regions where the traditional products are prepared [29,30]. This fact, as well as the decline of knowledge transfer and gaps in documenting the traditional know-how of local practices to manage the microbiota, ingredients, and fermentation process have favored the marginalization and disappearance of homemade fermented products. This phenomenon contrasts with the increasing interest in the nutritional value of ferments and their economic profits in markets, especially for the trendy fermented beverages, also called functional products, for instance, kombucha [29,30,31].

The human diet worldwide is vulnerable since it is mostly limited to 10 to 50 plant species that provide about 95% of the world’s caloric intake [32,33,34,35]. In contrast, ethnobotanists and anthropologists have documented thousands of edible species throughout the world. Only in Mexico, several studies have reported more than 2000 edible plant species, [36,37,38,39,40]. Among them, numerous wild species provide vegetables, fruits, nuts, tubers, and other edible products. However, little is known about the diversity of plant substrates that are employed for fermented products, their distribution, and if they are handled for specific fermentation purposes.

In 2010, the UNESCO recognized food as an intangible cultural heritage and included the Mexican gastronomy in the representative list of “Intangible Cultural Heritage of Humanity” [41,42]. However, the traditional fermented beverages are a neglected food group of the cultural heritage of the Mexican cuisine, and little is known about its conservation and biocultural status, permanence, or possible recovery. Several authors [43,44,45,46] have defined the traditional products considering the following criteria: (1) The key production steps of a traditional food product is performed in a certain national, regional, or local area, (2) the traditional food product must be authentic in its recipe, origin of raw material, and/or production process, (3) the traditional food product could have been exchanged locally for several years, available often through barter or only consumed by the family core, and (4) it is part of the gastronomic heritage. The tradition provides materials (raw substrates and tools) that allow groups to be rooted in their past and move and rebuild themselves in the present [43,44,45,46]. Therefore, it has been recognized that traditional products are dynamic.

In Mexico, a broad spectrum of traditional fermented foods, including beverages, is produced from different raw materials by Indigenous and mestizo people in different regions, the processing methods and microbiota varying among regions, localities, and producers [47,48,49,50]. A previous work by Godoy and collaborators [51] reported 66 types of fermented beverages, 4 of them registered only in historical books but not consumed anymore. Most of the beverages reported by Godoy are a combination of fermented agave sap and several fruits, known as curados. Pineda [52] described over 75 types of fermented beverages, including a few distilled for preparing spirits, listing numerous herbs, fruits, roots, and stalks that were used to infuse those beverages. Bruman [53] conducted an important work in the 1930s when he visited several regions of Mexico and recorded several traditional beverages; therefore, in this study we consider his work fundamental to characterize a previous distribution of the TMFB and to compare it with the present. The previous works help to identify historically important beverages prepared with wild or cultivated plants and to determine whether the plants or the traditional knowledge and practices have become rare or lost.

Several studies have been directed to characterize the microbial communities associated with some TMFB [47,48,49,50], their possible benefits to human health [47,48,49,50], and their biotechnological and functional applications as the probiotic activity or the production of antimicrobial activity [47,48,49,50]. However, to our knowledge, none of them have been under detailed clinical trials to corroborate their benefits for human health [47,48,49,50] However, few studies have been directed to characterize the rich legacy and diversity of fermented beverages as biocultural reservoirs of diversity of organisms and the variety of practices to elaborate a culturally accepted product.

The characterization of microbial communities of Mexican fermented products has been commonly performed by culture-dependent approaches. Early classifications of products were conducted based on the physiological and biochemical criteria and microscopic inspection [51]. Nowadays, molecular markers such as 16S and ITS have been used to accurately characterize pure strains of bacteria and fungi. Nevertheless, the scope for such approaches is limited, time-consuming, and the results might dismiss the identification of uncultivable microorganisms. Culture-independent approaches have been used in other studies; for instance, denaturing gradient gel electrophoresis (DGGE) has been used to characterize the microbial communities of TMFB such as **mescal** [47,48], **pulque** [54], and **pozol** [55]. Although culture-independent techniques give insights of the composition of microbial communities, these are also time-consuming and do not provide information on rare species.

More recently, some studies have used high throughput sequencing (HTS) techniques, such as Illumina MiSeq paired-end sequencing of barcoded polymerase chain reaction (PCR) amplicons, but these studies are still scarce in TMFB. However, studies with shotgun metagenomics have recently been conducted to characterize and infer the dynamics of microbial communities of **pulque** [56,57], **atole agrio** [58], and **tuba** [59]. A few studies have been performed under a polyphasic approach but most of these studies have been used to characterize the bacterial communities, while fungal communities have not been deeply studied. The study of microbial communities in fermented products is crucial since it allows for monitoring the processes and practices and gives insights of the distinctive autochthonous peculiarities of a product. Microbial diversity is a determinant for understanding the chemistry and nutritional properties for health, yield, and quality of the plant substrates employed for fermentation [60].

This review started with the assumption that the TMFB are the outcome of the synthesis of the vast biological and cultural diversity. In particular, we expected to identify a high diversity of TMFB, which is related to the similarly high cultural diversity represented by the number of Indigenous groups that are distributed throughout the different regions and ecosystems of Mexico. We expected that those more widely distributed and more studied groups from ethnobiological perspectives (the Nahua, Maya, Mixtec, and Zapotec in central and southern Mexico, as well as the Rarámuri, Seri, Pápago, or O’odham and the Tepehuan in northern Mexico [61]) are also those with more records of TMFB. The processes of cultural erosion have been documented in different regions, which have led to the loss of traditional cultural elements; we supposed these include TMFB. However, it is relevant to identify which ones and how much are endangered because of their decreasing availability or consumption. We, in addition, expected that those beverages based on substrates with a broader distribution and higher rooting would be the most frequent and more studied. Finally, we expected to identify a core of components of microbial communities in the diverse beverages since most of the TMFB pass through a lactic and alcoholic fermentation stage. The aims of this review are therefore to: (1) Characterize the diversity of the TMFB to provide an overview of the research on fermented beverages, the cultural groups that produce them, the plant substrates used, and the microorganisms identified; (2) visualize the spatial distribution of these beverages in the country; (3) identify their presence or absence in the foodscape and the status of conservation policies promoted by the Mexican authorities; and (4) identify the main trends of studies conducted on TMFB and the topics that are needed for a research agenda towards sustainable use of traditional ferments of Mexico. We emphasize the importance of the fermented beverages as reservoirs of biological diversity, and their relevance as Mexican biocultural heritage conferring identity to cultural groups as unique and diverse foodscapes. We, in addition, aspire to provide helpful information for designing strategies for conserving and recovering such a valuable heritage.

## 2. Materials and Methods

### 2.1. Literature Review

We conducted a search of peer-reviewed literature in Scopus, Google Scholar, Google, and the Web of Science databases to visualize the current state of research on TMFB. In addition, since numerous studies are not covered in these databases, we reviewed local journals, technical reports, books, and Ph.D. theses from regional universities of Mexico to complement the information. We also consulted references from gastronomic literature and governmental sources information. Through this search, we identified 328 peer-reviewed articles plus 197 other references related with TMFB. A search of peer-reviewed literature was performed with the following keywords: Mexican fermented beverages, traditional fermented beverages, **tepache**, **pulque**, **mescal**, **colonche**, **jobo** or **hobo**, **colonche**, **nawait**, **pozol**, **tejuino**, **tesgüino**, **piznate**, **taberna**, **cocoyol**, **tuba**, Mexican palm wine, **balché**, **xtabentún**. As these names are mainly in Spanish, we used the Boolean operators OR and AND. For example, in the Web of Science, the following search string was used: “Agave” AND “fermentation”, “tradicional” AND “bebidas”, “traditional” OR “ancestral” to improve the research parameters. We extended the search in local databases using keywords such as Mexican fermented beverages, bebidas fermentadas mexicanas, fermented beverages in Mexico, bebidas fermentadas en México, fermented products in Mexico, productos fermentados en México, **tepache**, **pulque, colonche**, **hobo**, Mexican wines, vinos mexicanos, **colonche**, **nawait**, **pozol**, **sidra**, **tejuino**, **tesgüino**, **taberna**, **cocoyol**, **tuba**, Mexican palm wine, and **balché**.

Most of the beverages recorded are wine-like beverages produced by the fermentation of several fruit species; however, we found numerous cases in which the producers use to add sugar cane alcohol or spirit beverages to confer specific flavors, and these beverages were not considered in our analysis. We found different names for beverages produced with **pulque** mixed with several fruits known as *pulques curados*; in this case, we only considered the name **pulque** for all of these beverages. In a broad sense, in this review, we only considered the main substrates for fermentation and secondary substrates employed during the fermentation process.

To characterize the current state of research on TMFB, the information of the title and the abstract of each article were used as inputs in the software VOSviewer 1.6.15 [62,63]. The construction and visualization of the resulting conceptual networks were performed with the full counting method with at least three occurrences of a term, and a consideration of 60% of relevance. The layout attraction and layout repulsion parameters were scored 1 and 0, respectively, the distance between nodes represents the connectivity among concepts, and the nodes size represents the number of mentions of the concept. The clustering resolution and minimum cluster size parameters were set to 1.25 and 5, respectively.

The information from this search was systematically stored in a database using Access with the following fields: (1) Fermented product name; (2) plant substrates (both scientific and local names; (3) microorganisms (both bacteria and yeasts, and the techniques employed to identify them, e.g., culture-dependent or culture-independent); (4) ethnic groups producing and using the beverage (the dosage of consumption, related customs, and rituals; whether it is still consumed or extinct); (5) sensorial features associated to beverages (sensorial attributes reported as sweet, acid, etc.); and (6) medicinal (if it has been reported to improve human health). Searches in the databases considered studies from 1960 to August 2020. In addition, to document the conservation policies over biological resources and traditional knowledge, we searched in governmental agencies databases.

### 2.2. Map Construction

A map was constructed to visualize the documented distribution of fermented beverages based on the data collected in the literature; the area of the municipalities identified was considered as the documented distribution area. Another map was constructed based on the estimated distribution regions proposed by Bruman [53]. In addition, to visualize the distribution of TMFB documented and that of the cultural groups, we considered the distribution of the Indigenous languages of Mexico reported by the Comisión Nacional para el Conocimiento y Uso de la Biodiversidad (CONABIO) [64]. The map design and analysis were made with the Qgis free software [65]. Finally, a map was constructed with the overlapped data of the documented distribution and Bruman’s proposal to visualize a potential area of distribution of the TMFB and the gaps that have not been documented.

### 2.3. Network Analysis

To visualize the uniqueness, diversity, and interaction of the substrates employed to produce fermented beverages, and the co-occurrence of specialist or generalist species in microbial communities, we performed an exploration through the bipartite network approach. The network’s theory offers powerful tools to describe complex communities and the distribution of species specificity within them. Moreover, this approach was performed with the microorganisms identified in the literature to each traditional fermented beverage. The data from the network structure were used as descriptors of diversity and uniqueness. The analysis was performed through the Rstudio software [66] with the igraph package [67].

## 3. Results

### 3.1. Traditional Mexican Fermented Beverages

Based on a previous review performed by Godoy and collaborators [51], 66 types of fermented beverages were registered. Nevertheless, most of them are beverages prepared with pulque whose main substrate is the fermented sap from *Agave* plants and the addition of fruits of native and introduced plant species. By the current review, we identified 16 names for traditional fermented beverages produced from several substrates such as seeds, stems, fruits, tree barks, fruit pulps, and sap. Stems are the dominant substrates used for preparing traditional fermented beverages. We identified 140 plant species used as main substrates for fermentation or as promoters of fermentation.

Through the peer-reviewed literature, we identified 10 traditional fermented beverages and we extended the number of studies based on the information from theses and the local literature, from which we identified 6 additional beverages; therefore, we documented a total of 16 fermented beverages. The most studied beverages are **mescal**, **pulque**, **tejuino**, **pozol**, **chorote**, **colonche**, **saká**, **sendechó**, **balché**, **atole agrio**, **pox**, **sambudia**, **tesgüino**, **tepache**, **tuba,** and **taberna** (Table 1).

Since we did not find recent studies on other beverages, we cannot confirm what is their actual status of production and distribution. Clearly, further field studies should be directed to document what is currently happening with these beverages. It can be visualized that the current research tendency is markedly directed towards the beverages grouped into the category of *Agave* spirits or *Agave* distillates referred to in this study as **mescal**, compared with the rest of the traditional fermented beverages in peer-reviewed literature.

**Figure 1:**
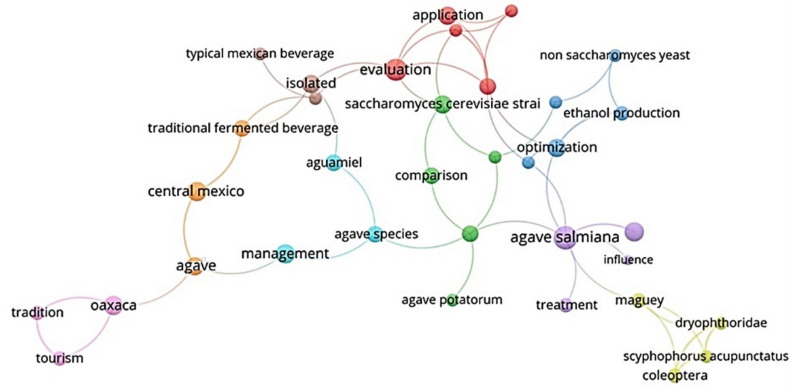
Clusters representing different research topics reported in the literature. The red cluster ● is related to biotechnological applications in traditional fermented beverages; the green cluster ● represents studies related to the mescal production; the blue cluster ● are those on the application of yeasts through the production of traditional fermented beverages; the olive cluster ● includes studies on pathogens affecting the development of *Agave*; the purple cluster ● comprises the research on environmental factors influencing the fermentation process; the light blue cluster ● are studies on management practices of *Agave* and sap extraction; the orange cluster ● indicates the reports on the presence of traditional fermented beverages in central Mexico, mainly **pulque** and **mescal**; the brown cluster ● shows the research that has been directed to maize beverages and the possible probiotic features associated with this microbiota; the pink cluster ● shows the importance of cultural groups of related to production of fermented beverages, but it is only related with mescal production.

The current state of research in peer-reviewed articles on TMFB can be visualized in the network of Figure 1, which displays nine conceptual clusters that highlight the main topics related to the beverages. Cluster (1) (in red) is related to the application, evaluation, and sensory properties of the fermented products. This cluster resembles the classical biotechnological approach from the early 1990s, most of the research being directed to improve the sensory properties of **mescal**. It also includes new trends related to the application of defined starter cultures. Cluster (2) (in green) is the **mescal** cluster, in which *Agave potatorum* appears as the most studied species, and *Saccharomyces cerevisiae* as the most commonly characterized microorganism. This cluster highlights the economic importance of **mescal** production, and the interest in improving the fermentation profile using several *S. cerevisiae* strains. Cluster (3), the yeast cluster (in blue), emphasizes the use of non-*Saccharomyces* yeast species to improve the aroma profile and optimize the ethanol production in **mescal**. Cluster (4) is the pathogens cluster (in olive) and refers to the effects of pathogens such as *Scyphophorus acupunctatus*, which mainly affect the *Agave* groups, decreasing the production of beverages related to these plants. Cluster (5) is the *Agave salmiana* cluster (in purple), which is mainly related to the influence of environmental factors in the development of plants and the fermentation process.

The following clusters are related to the most traditional beverages and human groups related to them: Cluster (6), the management cluster (in light blue), groups the management practices on *Agave* species, mainly those involved in the production of **pulque** and the implications of management of agaves for sap extraction. Cluster (7), the one of traditional fermented beverages (in orange), features the less common traditional fermented beverages prepared in central Mexico, those with *Agave* species such as **pulque** or **mescal** that occur mainly in central Mexico. Cluster (8), the maize group (in brown), mainly accounts for research directed to isolate and characterize the bacteria and yeasts associated to possible probiotic attributes in **tejuino** and **pozol**. Cluster (9), the cultural cluster (in light purple), groups studies related to traditions and tourism, mainly in the state of Oaxaca. It can be highlighted that this cluster is also related to **mescal** production.

The network shows that most of the current research is directed to study the **mescal** production process and its optimization. This is not surprising because of the national and international rising market boom of **mescal**. However, it is lower than research on tequila, which is the fourth largest export product of Mexico [142]. After **mescal**, **pulque** is the most studied beverage, particularly biotechnological and management aspects involved in its production.

### 3.2. Current State of the Conceptual Overview

The main microorganism referred to in the studies is *S. cerevisiae*, which is a common species in fermented products around the world and it is frequently found in alcoholic beverages. Surprisingly, it is possible to see in the network that the non-*Saccharomyces* species form a node; this is a trend around several industrialized beverages, such as beer [70], wine [143], and recently in cocoa fermentation [144], which looks for flavors and aromas in the final products. Research has been directed to characterize bacteria in the traditional fermented beverages, mainly because most of these beverages pass through a lactic fermentation stage, but perhaps the most common purpose is the isolation and evaluation of these bacteria to improve human health [145].

Two major trends can be identified in the current research on traditional fermented beverages in Mexico. The first one includes biotechnological approaches, which could be visualized in the first three clusters referred to above, which is related to the worldwide interest of the dairy industry to promote products with probiotic and prebiotic compounds [146]. This topic has been constitutively addressed in numerous research centers for several beverages around the world [147]. In the markets, there is an increasing demand for functional dairy products and healthy food [148,149] that pushes this major trend, which is reinforced by the search for options to improve nutrition in developing countries by using biotechnology [87,150]. In Mexico, this trend is clear in fermented beverages such as **pozol**, **pulque**, **tepache** and **tejuino**, whose microbiota and potential benefits to health have been majorly characterized.

The second research trend is related to the *Agave* beverages such as **pulque** and **mescal,** but also beverages such as **pozol**, which entails biotechnological approaches and the traditional management of sources of substrates, particularly *Agave* species. The rising market of **mescal** appears to be leading this trend, while in the case of **pulque**, the possible food functionalities appear to be the main drivers of the current research programs. It is possible to see the great cultural node related to **mescal** production, but few studies focused on the cultural groups in other beverages.

There is a huge gap in the research agendas on traditional fermented beverages. It is notorious the major interest that *Agave* products have but almost null on the rest of the traditional fermented beverages. As mentioned, there is high interest in biotechnological research programs specially to explore the central region of Mexico, which could be seen in Figure 4A. The remaining beverages are not only marginalized in markets, but also in the scientific agendas. Greater efforts to maintain them should be performed.

### 3.3. Plant Diversity Used in Mexican Traditional Fermented Beverages

**Figure 2:**
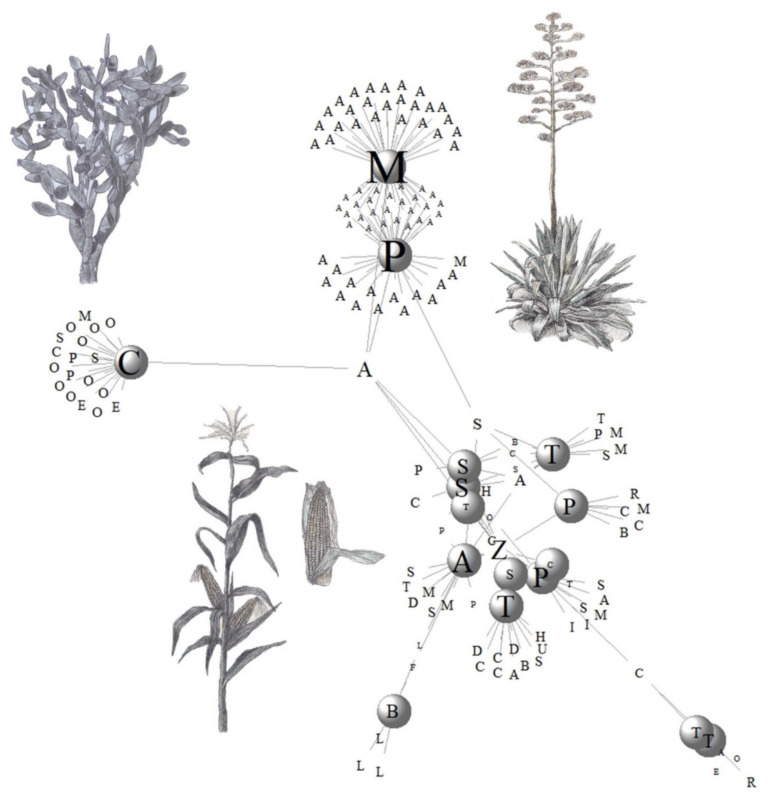
Network of substrates. Capital letters in the grey circles represent the TMFB, the size of circles and letters are related to the number of substrates employed; the letters without circles represent the species employed in fermentation, their size represents the number of uses in different fermented beverages. The M and P clusters are related to the substrates used for preparing **mescal** and **pulque**, respectively; the A represents the *Agave* species employed. The A in the middle of **pulque** and **mescal** cluster represents shared species, while in the external position represents exclusive agave species. The *C* circle is the **colonche** production, it is prepared with several cacti fruits, and shares *A. salmiana* for its production as other beverages in which the A is in the center of the network. The centered cluster is related to maize produced beverages (**pozol**, **tesgüino**, **tejuino**, **atole**, **chorote**, **saká**) in which *Z. mays* (Z in the middle) is the core substrate for these fermented beverages. The B cluster is the **balché** group, in which L letters refer to *Lonchocarpus* spp.; the T cluster is related to beverages prepared mainly by the fermentation of palm sap such as **taberna** and **tuba**. Species and beverages are listed in the Table S1. Illustration credits to Rosa Jeannine Xochicale Solís.

We identified 143 plant species used as main and secondary substrates for fermentation, as promoters of the fermentations process, and as additives to improve the shelf life or flavor (see Table S1). The network analysis reported a low connectance, a low number of links between the species and beverages analyzed, which elucidates the high specialization of ingredients, preparation, and assemblages of the beverages as can be seen in Figure 2, in which the clusters are relatively distant from each other (see also Table 2). This pattern is corroborated with the low niche overlap and the mean of shared partners in the network. The latter value also explains the low number of generalist species involved in the network; species such as *Z. mays (Z)*, *A. salmiana (A)*, *S. officinarum (S)*, and *Cinnamomum verum (C)* can be seen involved in the production of different fermented beverages. *S. officinarum* is a species frequently employed to strengthen fermentation as it is used as an external supply of sugars, commonly added as processed brown sugar. *C. verum* is used to add different flavors to the final product but not during the fermentation process. Cinnamon has been recorded to have antimicrobial and pathogens inhibitors properties, but it has not been reported on its use to avoid spoilage. Sugarcane and cinnamon are non-native species from Mexico but are strongly integrated into the traditional fermented beverages production as enhancers or additives.

The network of substrates of the traditional fermented beverages of Mexico is diverse (S = 5.21), with a minimal connectance, which indicates the uniqueness of most of the beverages with a high specificity in the substrates. The documentation and protection of those unique substrates should be attended to maintain and guarantee their production. The network allows for visualizing that maize is the most generalist substrate, which is not strange since maize is a keystone of the Mexican food systems and cultures and is prepared in great diverse ways.

#### 3.3.1. Cluster of Maize Beverages

Eight TMFB involve the use of maize as a main or secondary substrate, most of them are prepared with maize grains, including: **atole agrio**, **saká**, **tejuino**, **tesgüino**, **sendechó**, **chorote**, and **pozol**. **Pox** was usually prepared with maize stems as the main substrate, but sugarcane stem is more commonly used now. For processing most of these products, people employ specific races of maize; we identified 21 races used to produce them, and the beverages receive different names according to the maize race and locality where they are produced. Beverages produced with maize have a deeply and strong relationship with ethnic groups. Through this review, we identified that maize beverages are the most consumed by 21 cultural groups of Mexico.

**Tesgüino** is produced and consumed mainly by the Rarámuri or Tarahumara people and other cultural groups such as the Yaqui and the Wixarika, who live in northern Mexico [46,47,48,49,87,88,89,90]. This beverage is commonly mentioned as the Mexican beer or maize beer, and locally named batári by the Tarahumara. It is consumed for several cultural purposes related to celebrations such as weddings, funerals, rituals associated to practices of the agricultural cycle, baptisms, and for maintaining the cohesion of the communities in the celebrations called tesgüinadas [90]. It is a beverage mainly elaborated by women by fermenting sprouted maize kernels. About five days after germination, kernels are grounded and boiled for approximately 12 h, then cooled, and the mass obtained is placed in a container known as tesgüinera where fermentation takes place. It is a spontaneous fermentation where producers commonly add leaves and other parts of local weeds and trees such as *Stevia serrata*, *Chimaphila maculata*, *Datura meteloides*, *Hamaemelum nobile,* and *Cojoba arborea,* all of them identified with local names [90,91,92]. *Usnea dillenius*, a lichen, is used as a catalyst [90].

It is commonly assumed that **tejuino** and **tesgüino** are the same beverage since the fermentation core substrate in both cases is the grounded kernels of maize (a dough); however, the process, the plant species, and the varieties added, locations, and the cultural groups are different. **Tejuino** is a TMFB prepared with the dough of one variety of maize and the addition of brown sugar. It is mostly consumed in the states of Colima and Jalisco by mestizo people, and non-special containers are used for fermentation. The **atole agrio** is a similar beverage. It is the fermentation of maize dough, and no sugar is added. It has been recorded in southern Mexico and its consumption involves the use of different local races of maize among the localities.

**Pozol** and **saká,** are non-alcoholic fermented beverages from the Mayan region, prepared with dry corn kernels, boiled with calcareous stones and then grinded into a dough. This is a process of nixtamalization that confers a particular consistency and flavor to the dough and adds nutrients such as calcium oxide and others that become bioavailable [54,81,151]. This maize dough can be prepared with several maize races available in the region. For both beverages, the process is clearly similar, but **pozol** is more common among the mestizo communities and **saká** is associated to Indigenous communities in religious contexts. For its production, maize dough is stored into small balls in banana leaves until fermentation, a process that varies depending on the environmental conditions and the producers’ preferences. The dough balls are dissolved in water and consumed as a daily beverage [54,81,82,83,91,92,151]. In different locations, **pozol** dough can be mixed with batata, toasted cocoa, coconut, cinnamon, or vanilla [72,73,74,75,76]. These beverages are considered a source of nutrients and an essential food in southern Mexico, where its consumption is associated with traditional ceremonies, or consumed as a daily drink. It is also consumed as remedy for gastrointestinal illnesses in some localities [54,76,77,78,79].

**Sendechó** is a beverage that can be prepared with several maize races (five reported), depending on the producers’ preferences, so its final color might vary from light red to purple, light yellow, white, and black. Through the germinating grains step, *Buddleja americana* is mixed with the grains [48,49]. **Pulque**, called ixquini in the region, is added as a starter inoculum [137,138]. The production process is similar to those maize beverages mentioned above; sprouted maize grains are ground, in this step chili peppers could be added [138], and then a maize dough is boiled with water, it is filtered with a cloth, and then spilled in a clay pot where *Agave* sap or fermented sap can be added as a starter of fermentation. It is produced in areas close to Mexico City and is still consumed by the Mazahua, Otomi, and mestizo groups for several cultural purposes.

#### 3.3.2. Cluster of Agave Beverages

The cluster of *Agave* beverages is a highly diverse group, with 68 agave species involved in the fermentation of **pulque** and **mescal** as the main substrate to produce both beverages. The fermented sap of *A. salmiana* is the most frequently employed to produce **pulque** and it is also added as an inoculum to other beverages. Although *Agave* is a diverse group, we recorded low connectivity among its components, which suggests unique fermented products. Since species and varieties of *Agave* are not explicitly recorded in some studies, we might be underestimating their distinctiveness; there are several local names for the local varieties, suggesting high variation, but unfortunately, their taxonomic identity has been insufficiently studied and indicated.

**Mescal** appears to be the predominant beverage of *Agave* throughout the Mexican territory. It is in fact the beverage that employs a major number of species to get a final product, most of them are locally distributed and different species are often mixed to produce a unique sensorial profile. We consider **mescal** as a TMFB because most of the species employed for its production are endemic to Mexico, and most of them wild but some have gone through a domestication process or are under different levels of domestication by people of this country. Moreover, because these products are locally and/or regionally produced, their production is mostly unique for each producer, are part of the local gastronomic heritage, and include local and foreign technologies as mentioned before [45,46,47,48]. This group is highly variable in the production process among regions and producers, using different substrates, fermentative spaces, types of containers, and fermentation techniques. In fact, local names for mescal spirits are diverse and depend mostly on the region and substrate employed. The basic fermentative substrates are the cooked *Agave* stems and foliar bases whose products are then distilled; however, the species used are numerous and variable among the regions.

As mentioned, we did not consider other plant or animal products that are often mixed with pulque consumption which are generally called pulques curados [93,94,95]. We did not consider them because the main substrate is the fermented agave sap and fruits are added after **pulque** is produced. Moreover, we did not consider the species that are used as tools, such as *Lagenaria siceraria*, to store or transport the sap of *Agave* species. However, the number of *Agave* species employed as main substrates for **pulque** production (42 species) is high. **Pulque** results from the fermentation of *Agave* sap, and it has been recorded as spontaneous fermentation [91,92], but also as the product of using inoculums, which have been characterized in different regions [23,93,94,95,96]. **Pulque** preparation starts with the collection of the agave sap called aguamiel directly from the agave stem, which is mostly performed under non-sterile conditions [95,96]. The fermentation process varies depending on the agave species, the quality of the sap, the region, and other factors. There is not one single type of **pulque** because of the plethora of practices, species, and conditions for its production. These practices might change the fermentation process in time, the assemblages of yeasts and bacteria, and the final sensorial attributes.

**Pulque** has been used historically for festivities, ceremonies, agricultural rituals, births, and funerals [93]. There is a specific regulatory system for **pulque** in Mexican law (NMX-V-022.1972), which defines quality standards and sensorial attributes. In addition to the health benefits of **pulque** or its biotechnological applications, the diversity of plants employed and the practices performed reflect a historical know-how developed by humans in interaction with their local environments. However, this production system has been marginalized.

#### 3.3.3. Cluster of Cacti Beverages

**Colonche** is a traditional fermented beverage that can be prepared with fruits of at least 17 cacti species. **Colonche** is the common name of this beverage in the region of the Altiplano central region of Mexico, where it is mainly prepared with several *Opuntia* species, although the most common and preferred by the producers and consumers is that prepared with *O. streptacantha* [22,50]. **Nawait** is another common name for a fermented beverage mainly produced by the fermentation of *Carnegiea gigantea* fruits, whose distribution covers the Sonoran Desert in northern Mexico and the southwestern U.S.A. [52]. In this region, a **colonche**-like beverage is also prepared with *Pachycereus pringlei*. In the Tehuacán-Cuicatlán Valley in south-central Mexico, **colonche** is known as nochoctli or pulque rojo. There, the fruits of several cacti are employed to produce this fermented beverage, the most common are those of *Pachycereus weberi*, *Escontria chiotilla*, and *Stenocereus* spp. This beverage is produced in different seasons depending on the availability of the substrates. For instance, in south-central Mexico, colonche is produced in April–May and in August–September, when fruits of the different species of columnar cacti and *Opuntia* used to produce it are available. The production and fermentations practices of **colonche** are variable in the different regions [22]. In most regions, fermentation occurs spontaneously; nonetheless, inoculums are employed by some producers by the addition of pulque from *A. salmiana* or by using inoculums from previous fermentations of cacti fruits. Most of the fermentations occur in clay pots continually used and that have been maintained by several generations of the managers [22].

#### 3.3.4. Cluster of Palm Beverages

The beverages **tuba** and **taberna** form the palm cluster. These are prepared by the fermentation of the sap extracted from different palm trees, similarly as it is carried out with other palm wines consumed around the world, such as legmi in Africa, or kallu in southern India, as well as several Asian palm wines [98,99]. The main differences of these beverages are the palm species and the palm parts from which sap is obtained [99,100]. These beverages were adopted in Mexico by the Philippine influence during the Spanish colonial period and are currently produced in the Pacific coastal zones of Mexico. The species used for producing **taberna** is *Acrocomia aculeata* known as coyol, and it is produced in localities of the southern state of Chiapas [100,139,140,141,152,153,154,155]. The production occurs by the deliberate cut of the shoot apical meristem of the palm tree, then by making a cavity where sap is accumulated and fermented. The cavity is covered to avoid contamination by insects and then the sap is collected and consumed fresh. Sap for preparing **tuba** is obtained from the inflorescences of *Cocos nucifera*, which is collected and then stored in plastic containers, although the iconic containers are those manufactured with *L. siceraria* fruits, which are called bules [98,99,100,139,140,141,152,153,154,155].

#### 3.3.5. Balché Cluster Beverages

**Balché** is an exceptionally important TMFB since it is the sacred drink for the Mayan, consumed in several ceremonies. It is the result of the fermentation of bark from several *Lonchocarpus* species, among them *L. punctatus* or *L. violaceus*, and *L. longistylus*, which is mixed with honey (either from *Apis mellifera* or melliponini bees). It has a particular pink color and sweet taste, and has been consumed since pre-Hispanic times [84,128,129,130,131,132]. To produce this beverage, some producers boil the bark of the tree to remove the compounds that confer a bitter flavor, thus the bark releases its characteristic color and fragrance. Then, it is dried to later be boiled with virgin water, which is collected from cenotes or rivers. After that, the bark, honey, and water are mixed and fermented spontaneously in a hole made inside a tree; later, the hole is sealed with banana or palm leaves for two or three days. Hitherto, *S. cerevisiae* has been the only microorganism recorded in the fermentation of this beverage [133], but more studies are clearly needed.

#### 3.3.6. The Cluster of Tepache and Sambudia

**Tepache** can be prepared with at least 10 plant species as a substrate. Nowadays, in central Mexico the most common substrate is pineapple (*Ananas comosus*), but its etymology in the Náhuatl language derives from tepitl, a beverage made with maize in the past and in few localities [49,50], which suggests that this beverage is a derivation from an ancient maize beverage. In western Mexico, **tepache** is also produced by the fermentation of other fruits that belong to the Bromeliaceae family, such as *Bromelia karatas*, known as tumbiriche or timbiriche. It can also be prepared with fruits of plants introduced into Mexico such as apple, orange, and guava [117,118]. In the case of **tepache** prepared with pineapple, the process starts by peeling the infrutescences and adding the rind into a wooden container known as tepacheras, then brown sugar is added. It is a spontaneous fermentation where the microbiota is mainly associated with the sorosis epidermis [49,50]. Production of **tepache** with other fruits can be fermented in plastic containers; this beverage is commonly homemade, and its quality varies from kitchen to kitchen.

**Sambudia** is a beverage prepared with several substrates, including maize, rice, and barley. **Sambudia** is the name used for different beverages produced in the state of Mexico; a first way to produce it is using pineapple peel as a substrate [50,134,135,136] through a process similar to that of **tepache**. A second way to prepare it is with ground grains of rice, or barley; in this case, cinnamon, cloves, pepper, roasted and ground maize leaves, and pulque are added in a container; brown sugar is added to sweeten the mixed elements and the mixture is fermented for at least one day [136]. Moreover, it can be prepared with the fermentation of *B. karatas* fruits by adding pineapple peel, maize leaf, and ground maize. Fermentation occurs by inoculating the remnants of past fermentations in the containers, which are clay pots called sambudieras [134].

### 3.4. Traditional Fermented Beverages as Dynamic Systems: The Addition of New Substrates

The historical records indicate that practices and techniques for manufacturing fermented foods evolved independently in every hemisphere and were developed based on the resources available in the local environments [12]. In Mexico, the fermented beverages, practices, and techniques are applied on the three main groups of substrates referred to above. However, the inclusion of foreign species or technologies to diversify the diet are not an exception in the Mexican cuisine. Therefore, rather than cultural erosion, the contact with new species, ingredients, and techniques of the Old World appears to have enriched the Mexican fermented beverages. These historical processes support a diversification hypothesis [101].

As humans migrated from region to region, food cultures and production practices moved as well [12,101,102,103,104,105]. Although the most common plants used as main substrates to produce fermented beverages are maize, agaves, and columnar cacti, the inclusion of new species replaced some substrates. A clear example is the traditional fermented beverage **pox** prepared with the stems of *Z. mays* nowadays, mainly prepared with *S. officinarum* stems [53], but also the several beverages with added brown sugar from this crop originating in Southeast Asia [106]. *C. verum*, also originated in Asia [107,108,109], is commonly used as an additive to flavor several Mexican beverages.

The fermentation of numerous palms sap is common in countries of Asia and Africa [96,98,99,100,101,102,103,104,105,106,107,108,109,110,111] and, in Mexico, **taberna** and **tuba** are examples of the adoption of new technologies and instruments, including distillers for the Philippine coconut spirits distillation technique [112,113,114]. Nowadays, there is a debate about possible pre-Columbian distillation, but the fact is that the Philippine distillation technique is commonly used in several localities for producing **mescal** and other beverages [115].

### 3.5. Traditional Knowledge and Microbial Communities in Fermented Beverages: How Do Traditional Fermenters Promote Microbial Reservoirs and Microbial Diversity

The most common practice to promote fermentation worldwide is the inoculation of a substrate with starter cultures. For instance, in Korea, soy sauce is prepared using meju (solely fermented soybeans), whereas in Japan and China it is prepared using koji (a fermented mixture of soybeans, wheat flour, and wheat) [156]. Similar examples exist around the world and, recently, the use and characterization of starter cultures has been a main trend of research that could be used to improve fermentations and prevent the possible risk of spoilage [157,158,159]. This is, for instance, the case of traditional and industrial production of wines [160,161,162,163,164]. However, there are still few studies identifying and characterizing traditional starter cultures for traditional products in Mexico.

Through this review, we identified names such as tibicos, castaña, xinaiste, jinaiste, zinaiste, el pie, el pie de pulque, asiento, ixquini, semilla, xaxtle, and nancle among the most frequent names that fermenters give to those mixed starter cultures for traditional fermented beverages. The study and characterization of these microbial communities have only been scarcely covered in studies of beverages such as **mescal** [97,165], **pulque** [61,166,167], and recently in **atole agrio** [21]. This technique has been used for years and the methods to prepare them should be considered in further studies.

A commonly accepted assumption in traditional fermented products is that spontaneous fermentations have inconsistent or heterogeneous quality outcomes. However, fermentation managers procure to simplify the diversity of the environmental conditions and practices to decrease such heterogeneity. The practice of selecting fermentative environments or controlled facilities is common; for instance, people procure fermenting inside the house or special areas to control the external environmental heterogeneity in light incidence, temperature, external contaminants, or pathogens. Moreover, there is a cultural selection of the person who performs the fermentation and the substrate’s quality [22,23,97,163,164]. A commonly overlooked aspect is how the microbial communities of starters are selected, prepared, conceived, stored, and used by local fermenters looking for the most favorable composition to improve the quality of their final products. Documenting such a process would give insights about how these communities of microorganisms are managed.

The interrelationships between the native microbial strains, substrates, techniques, tools, and fermentative environments of TMFB have been little studied. We identified studies that characterized the clay pots or tinas de fermentación for pulque [164] and **tesgüino** [85]. In fact, for **tesgüino** clay pots, fermenters use the specific name of tesgüineras and if the clay pot is broken, the pieces are trembled inside a new one, thus maintaining the microbiota from the old one. Nevertheless, few studies have attended to the importance of these containers as reservoirs of microbiota and their relevance in the fermentation process.

#### How Diverse Are the Inconspicuous Microbial Environments?

As mentioned, most studies on microbial environments in fermented beverages have been conducted with culture-dependent methods, isolating the most common microorganisms, among them *S. cerevisiae*, as the main microorganism responsible for alcoholic fermentation. These are invariably present in **mescal** and **pulque**. Nevertheless, for maize fermented beverages, the isolation and characterization of microorganisms have been directed to bacteria to characterize the possible probiotic functions of lactic acid bacteria (LAB) and, more recently, their function as a source of nitrogen fixation bacteria [163].

**Figure 3:**
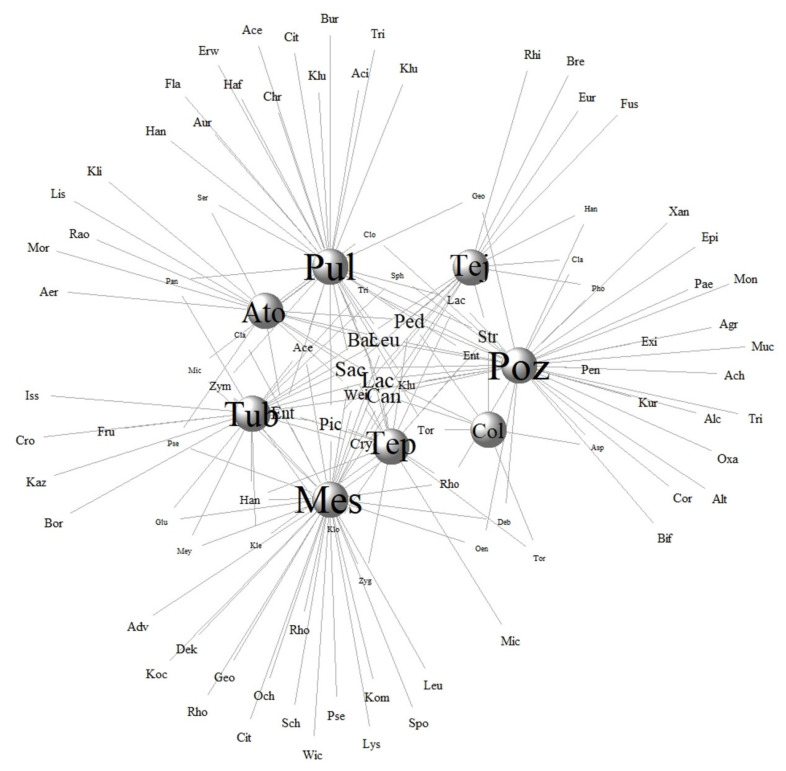
Network of microorganisms. Grey circles represent the names of the TMFB, Tep is for **tepache,** Col is for **colonche**, Mes is **mescal**, Ato for **atole agrío**, Pul for **pulque**, Tub for **tuba**, and **Tej** for tejuino. The size of the letters represents the number of microorganisms recorded for each beverage. The letters in the middle represent the microorganisms that are shared commonly in the studied beverages. *Saccharomyces* (Sac), *Lactobacillus* (Lac), and *Candida* (Can), among others, are common shared microorganisms. **Mescal**, **pulque**, and **pozol** are the most studied beverages in which a major group of microorganisms has been recorded. **Colonche** displays the lowest connectivity due to the few studies on this beverage. The genera of microorganisms and the names of beverages are displayed in the Table S2.

We found that only 10 beverages have been studied to characterize their microorganism communities, identifying 255 species of microorganisms. **Pozol** (Poz), **pulque** (Pul), and **mescal** (Mes) are the beverages with more species recorded (Table S2). This could be partially explained because a larger number of studies have been carried out on these beverages as also shown in the conceptual network (Figure 2). The network analysis reveals low connectivity that may be explained because there is high specificity in microorganisms in some of the beverages (Figure 3). In addition, it shows numerous links per species since genera such as *Lactobacillus* (Lac), *Bacillus* (Bac), and *Saccharomyces* (Sac) are shared among most groups of beverages. The network values are shown in Table 2.

Through the nested analysis, we can see that **mescal**, **pulque**, **atole agrio**, and **pozol** are the beverages with the highest number of microorganism species identified, while **colonche** is the beverage with fewer genera identified. In general, six modules of microorganisms in TMFB were detected: (1) **mescal**; (2) **pulque**; (3) **atole agrio**; (4) **pozol**; (5) **colonche**, and (6) palm and **tepache**. This analysis shows that 99 species occur in the most studied fermented beverages, which might be the generalist core of microorganisms intervening in these fermented products. Interestingly, with this analysis, we identified that **pozol** has 24, **mescal** 23, **pulque** 22, **atole agrio** 10, and **colonche** 2 genera with low shared similarities with other beverages, and those could be specialist genera in each traditional beverage. Nevertheless, as the production practices and substrates vary for each event of production and ferment type, further analyses are needed for more precise information.

Forthcoming technologies such as CRISPR/cas9 [167,168,169,170,171,172,173,174] or the improvement of specific strains [115,156] could play important roles to improve these products as it has happened with yeast from *S. cerevisiae* in the fermentation of several products [172,173]. Nevertheless, careful studies should be performed about how and when these microorganisms are or can be intentionally used in these beverages because there is not a clear panorama about the ecological implications of using these microorganisms in replacing local native microbiota.

### 3.6. Uses of Fermented Beverages by Human Cultural Groups and Future Directions

Mestizo people produce and consume most of the TMFB, mainly those prepared with maize and agave as the main substrates. This could be explained because mestizo people are the most numerous in Mexican society and have recovered Indigenous traditions; also, because maize plays the key role in Mexican gastronomy, it is widespread, and fundamental in the local communities’ nutrition [175,176]. These facts also explain that maize is the most generalist substrate of TMFB and the key role of these beverages in social cohesion, festivities, and ceremonies, such as tesgüinadas [177,178]. Likewise, many of the TMFB that have been recorded are used as medicine to prevent diarrhea, reduce infections, constipation, and, in general, to improve health. These beverages are currently part of daily life food, refreshing beverages during the working hours, and part of local ceremonies.

Indigenous cultural groups are interested in looking forward and renewing their connections with their lands and cultural heritage and recovering their traditional foodways to regain cultural strength and personal and community health [30,179]. This could be visualized in numerous forms of research that have identified how communitarian leaders and elders have encouraged youth to learn about harvesting and how to prepare their traditional foods around the world. Combining knowledge from different sources and epistemic systems is necessary to understand the diversity within and across ecological, social, and cultural systems, which are important factors underpinning conservation and natural resource management strategies [179,180,181]. This should be a particular concern in the case of traditional fermented beverages around the world, not only in Mexico.

**Figure 4:**
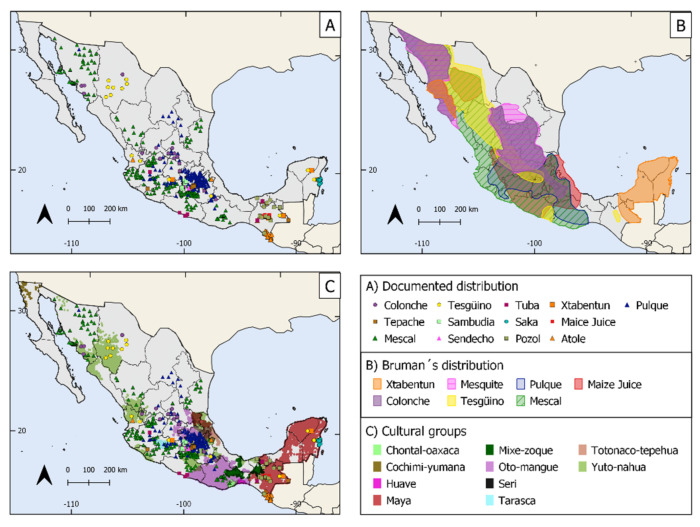
Maps of distribution of TMFB: (**A**) documented distribution of the TMFB; labels are indicated in the low right rectangle; (**B**) distribution proposed by Bruman; (**C**) distribution of TMFB according to the current review and cultural groups grouped by language.

It is important to highlight the marked level of segregation of the Mexican Indigenous people, which has been verified by numerous anthropological and socio-demographic studies based on synthetic demographic and poverty indexes [182,183,184,185]. Figure 4C shows a clear correspondence of TMFB with cultural groups. Most of them correspond to the mestizo group in the central region of Mexico. It should be considered that Indigenous people are not a homogeneous sector. The censuses of the Mexican population report gradients from monolingual to bilingual speakers [185], and numerous studies show that most Mexican people have extraordinary mixtures of Indigenous and non-Indigenous cultural aspects. However, it is generally recognized that Indigenous people are one of the sectors living in extreme poverty with no access to education and health services. The lack of monetary income and household goods and social marginality are factors eroding Indigenous cultures, pushing people to migrate from their original areas to cities, where Indigenous languages and culture are often discriminated [185]. This process endangers the maintenance of general culture and the transmission of the traditional knowledge on the production of fermented beverages.

Efforts should be directed to characterize and document aspects of TMFB, such as their uniqueness, human cultural specificity, their heritage status, the dietary patterns, their role in cultural identity, practices in the agricultural production of raw materials for fermentation, dishes and gastronomic innovation, preparation techniques, recipes, food traditions, symbolic dimensions, and material aspects such as utensils and dishware, among other biocultural topics. In addition, promotion activities would be relevant. For instance, promoting events in the communities, sponsored through schools, councils, and cultural centers, can be effective to illustrate, highlight, and demonstrate the values of traditional food systems [186,187]. These activities could help to maintain this marginalized biocultural heritage.

### 3.7. Reviewed Distribution of the TMFB

We compared the distribution of TMFB documented by Bruman in 1993 (Figure 4B) with information documented in this review (Figure 4A). We overlapped both maps to visualize the localities where these beverages could be produced nowadays and, accordingly, should be attended as priorities in further studies (Figure 4). It can be noticed that Bruman reported 7 beverages and we found 15, adding specific sites of **tepache**, **sendechó**, **sambudia**, **atole agrio**, **saká**, **tuba,** and **pozol** distribution.

The most striking result is the current absence of mention of a traditional fermented beverage made with mesquite pods reported by Bruman and a beverage named **bingarrote** or **bingui** from central Mexico, reported by Pineda [52], which was prepared with underground cooked and then crushed maguey stems; this material was placed to ferment into a pulque container, and afterwards the fermented liquid was distilled in an alembic. The ferment is called **binguí** or **benjuí** and the spirit **bingarrote**. It is a sort of culinary hybrid between pulque and mescal, but it is unclear which agave species was used. This information could be crucial to recover this apparently lost food, which could be a lost knowledge to humanity [188,189,190,191].

A first sight of maps in Figure 4 suggests that there has been a dramatic reduction in the production and consumption of the traditional fermented beverages, since Bruman defined areas in the early 1990s until the current research. However, it is important to emphasize that Bruman’s methodology was mainly based on his expert knowledge and data that he collected through fieldtrips. We identify that the locations recorded in our review represent shorter areas due to the following reasons: (1) there is a gap in the research on uncommon traditional beverages contrasting with that on **pulque** and **mescal**, (2) there is a systematic oversampling in the same localities for most of the studies; for instance, five **pozol** studies are performed in the same locality and this fact does not allow characterizing the potential area of this beverage, and (3) there has not been regular monitoring of the production of some of these beverages, and, consequently, it has not been attended before.

We therefore propose that deeper studies are still necessary to identify, characterize, and monitor the actual and potential production areas of TMFB. This task will require fieldwork, information about the distribution of the substrates related to each beverage, and a careful characterization of localities where it is produced, using approaches from ethnobiology and ethnography that could bring insights about the possible processes of cultural erosion and the effects of market pressures and cultural discrimination over these marginal products. Such studies could also be the way to promote maintaining or the revival of these products [28,192]. The loss of traditional food systems will result in decreasing culture-specific food activities, thus influencing the decrease of dietary diversity [193]. Moreover, TMFB could be part of touristic development, reliable small-scale food markets, and local health strategies, but most of them remain unexplored and some others are in danger of extinction.

### 3.8. How Are We Attending the Maintenance of This Intangible Biocultural Heritage?

In recent years, the awareness to protect traditional relationships between humans and environments has promoted the development of systems of legal protection and biocultural management around the world [194]. Numerous challenges have arisen to protect genetic resources, intellectual property rights, and natural resources, among other important topics related to traditional local knowledge [195,196]. Studies have been directed to document the local knowledge systems in several countries to define what is local and global, traditional, and Indigenous, and the authenticity and origin, as well as different ways to interact with the environment and how to attend to environmental crisis issues [197,198]. Traditional food systems can play a key role in the strategy to combat malnutrition while ensuring sustainable development [199]. The Food and Agricultural Organization (FAO) now recognizes that food items such as fermented products must be considered important in areas where malnutrition is evident. Use and conservation are commonly associated issues; conserving traditional food is a powerful way to conserve biocultural heritage.

Food systems are part of complex bodies of knowledge, most of them constructed empirically [200] and transmitted in multiple verbal and non-verbal ways, such as know-how or learning-by-doing [200], and fermented products are not exceptions. These products have been embedded as part of the daily lives of many people, including those currently marginalized rural or Indigenous groups [201]. The diversity of fermented products is an outstanding reservoir of genetic resources that has high potential to obtain secondary products such as extracts, enzymes, dyes, and others compound that can be involved in global markets and could help to solve problems such as hunger [202,203] and poverty and may play a key role to reinforce cultural identity [12]. Nevertheless, several cases of the commodification and commercial use of local knowledge have been recorded, which commonly decontextualize and make inappropriate retribution, and contribute to disarticulate local communities [204,205,206,207]. Therefore, analyzing the conditions for fair trade are indispensable when analyzing sustainable ways of using these traditional resources and products. This is a particular concern in relation to the industrial **mescal** production, for instance.

The Mexican government signed the Nagoya Protocol in 2011 and was ratified in 2012. This is the major international regulation system about access to genetic resources and fair and equitable sharing of the benefits derived from their use, which emerged from the Convention on Biological Diversity. However, it was not until 2014 that the Nagoya Protocol was enacted as a supreme law in article 133 of the Mexican Constitution. The main aim of this protocol is favoring incentives for the conservation of biological diversity, the sustainable use of its components, and the prevention of misappropriation of genetic resources and traditional knowledge on them, which is relevant for a bioculturally mega-diverse country such as Mexico.

Since Indigenous peoples hold traditional food system knowledge, interinstitutional and intersectoral initiatives would make more significant contribution to increase the potential of these resources, but local people should be protagonists. The documentation of traditional food systems is urgent since knowledge of food harvesting and preparation is rapidly disappearing. It is therefore crucial to enhance the public policies to support the maintenance of this traditional knowledge.

The conservation of species and varieties involved is equally important. Cultural historians, ethnobiologists, and the general public should become aware of the distinctive varieties of plants used as substrates for fermentation, their conservation, and recovery. For instance, the varieties of *Agave rhodacantha* classified by the Mayo people as San Antoneña and El Chino were used in the fermentation on an agave beverage called **yocogihua** in southern Sonora and northern Sinaloa. Most commercial production of mescal in this region blinked out during the Ley Seca that began in Sonora in 1916, but remarkably the persistence of these varieties was documented off and on from 1888 to 1965 when the last plantation of them was closed. Nevertheless, the Mayo who worked in the plantation rescued or dispersed them to rancherias neighboring the plantation in Masiaca, Sonora, thus several fencerow plantings of *A. rhodocantha* persist nearby, even though it is otherwise rare except for one other location in Sonora. The recovery of these cultivars and possible reintegration into the contemporary production of culturally distinctive fermented beverages in Sonora is now being undertaken [208,209]. As mentioned, **bingarrote** and **bingui** remain used for an analogous beverage today that is rarely produced in the state of Guanajuato where *A. salmiana* varieties are still in use for both pulque and mezcal production [209]. Identifying through community elders which varieties or species are currently associated with sporadic **bingarrote** and **bingui** production near San Miguel de Allende could help to rescue both the plant genetic resources and the traditional cultural practices historically linked to them.

For local knowledge associated with microorganisms, the situation might be overly complex and difficult to attend, as certain microorganisms are not cultivable and not easy to identify. The lack of a microorganism’s accessions in collections, the low characterization of the selection mechanisms, and the implications of this selection over genetic and phenotypic traits in microorganisms can make the establishment of protective laws or conservation policies on this topic challenging. To understand the crucial roles of microorganisms on the TMFB process, we need to know their ecological niches, population dynamics, and relationships between microbiome and environments and microbiome and the selection process.

## 4. Conclusions

Mexico has a unique gastronomic culture characterized by its multiethnic, high biocultural diversity and long cultural history. Biological diversity is used in traditional Mexican cuisine, and, undoubtedly, Mexican fermented beverages are intimately associated with plant products that have been managed and domesticated in Mexico, as it can be seen in the relevance of fermented beverages based on maize, agave sap and stems, or cacti fruits. However, this is also the case of dozens of wild species that are involved in traditional beverages. Foreign technologies, tools, and plants have been integrated into the Mexican foodscapes, and this fact has promoted a diversification of beverages and a diversification of sensory qualities acceptable to a wide range of palates. TMFB are clear examples that traditional knowledge is a dynamic process that is constantly changing, adjusting, and evolving.

Traditional Mexican cuisine is an outstanding reservoir of genetic resources such as plants, animals, fungi, and, generally less considered, microorganisms such as yeast and bacteria which could play a relevant role for future nourishment applications. The TFMB are reservoirs of cultural diversity, practices, and worldviews of communities. However, studies have focused mainly on the biotechnological applications and on beverages of the *Agave* group, leaving apart other beverages that are rarely studied, thus limiting the action to combat the endangered permanence of these products and the local food systems. The study of fermented beverages under transdisciplinary approaches is fundamental to provide information to construct regulatory frameworks or proposals to protect the diversity that TMFB involves in maintaining the complex interactions between humans, plants, and microorganisms. We expect that these examples demonstrate how biocultural conservation and restoration can be tangibly integrated into the protection and promotion of the Mesoamerican gastronomic patrimony recognized by the UNESCO and the routes of research needed to achieve it.

## Figures and Tables

**Table 1:** Main traditional Mexican fermented beverages. Main substrates for fermentation, microorganisms described, and cultural groups associated to its production.

Beverages	Main Substrate	Main Microorganisms Recorded in the Literature	Cultural Groups Associated	Literature
**Pozol**	*Zea mays* (grains)	**Bacteria***: Aerobacter, Acetobacter, Achromobacter, Agrobacterium, Alcaligenes, Bacillus, Bifidobacterium, Clostridium, Enterobacter, Enterococcus, Escherichia, Exiguabacterium, Klebsiella, Kosakonia Lactobacillus, Lactococcus, Leuconostoc, Paracolobactrum, Pediococcus, Pseudomonas, Propionibacterium, Streptococcus, Weissella.* **Yeasts**: *Candida, Cyberlindera, Debaryomyces, Kluyveromyces, Galactomyces, Meyerozyma, Pichia, Rhodotorula*, *Trichosporon*. **Fungi**: *Cladosporium, Monilia, Mucor, Phoma, Penicillium.*	Chol, Chontal, Lacandon, Mam, Maya, Tojolabal, Tzeltal, Tzotzil, Zapotec, Zoque, Mestizo	[52,53,54,55,61,68,69,70] [71,72,73,74,75,76,77,78,79]
**Atole agrio**	*Zea mays* (grains)	**Bacteria**: *Acetobacter, Aerococcus, Bacillus, Enterococcus, Clostridium, Lactobacillus, Lactococcus, Leuconostoc, Pediococcus, Streptococcus,* Weissella. **Yeasts:** *Candida*, *Cryptococcus*, *Clavispora*, *Debaryomyces*, *Hanseniaspor, Pichia Saccharomycesa.* **Fungi:** *Aspergillus, Fusarium, Penicillium.*	Maya, Mazatec, Nahua, Purepecha, Totonac, Tzeltal, Tzotzil, Wixarika, Mestizo	[58,72,73,80,81,82,83]
**Saká**	*Zea mays* (grains)	ND	Maya, Tzotzil, Tzeltal	[84]
**Tejuino**	*Zea mays* (grains)	**Bacteria**: *Acetobacter, Bacillus, Brochothrix, Chyseobacterium*, *Kurthia, Lactobacillus, Leuconostoc, Pantoea, Pseudomoonas, Strotococcus, Weissella*. **Yeasts**: *Candida, Galactomyces, Lachancea, Meyerozyma, Saccharomyces, Wickehamomyces.***Fungi**. *Aspergillus, Penicillium*	Mestizo	[85,86]
**Tesgüino**	*Zea mays* (grains)	**Bacteria**: *Bacillus, Lactobacillus, Bacillus, Leuconostoc, Pediococcus, Streptococcus*. **Yeasts**: *Brettanomyces, Candida, Clavispora, Cryptococcus, Kluyveromyces, Lachancea, Metschnikowia, Meyerozyma, Pichia, Saccharomyces, Wicherhamomyces*. **Fungi**: *Aspergillus, Penicillium.*	Guajiro, Pame, Pima, Tarahumara, Tepehuan, Tubar, Wixarika, Yaqui, Zapotec	[46,47,48,49,87,88,89,90,91,92]
**Pulque**	*Agave* spp. (sap)	**Bacteria**: *Acetobacter, Acetobacterium, Acinetobacter, Acrobacter, Adlercreutzia, Ardescatena, Bacillus, Commensalibacter, Citrobacter, Cellulomonas, Cellulosimicrobium, Chelativorum, Chryseobacterium, Chryseomonas, Clostridium, Comensalbacter, Corynebacterium, Devosia*, *Dysgonomonas*, *Enterobacter, Erwinia, Escherichia, Euzebia, Flavobacterium, Fructobacillus, Gluconobacter, Hafnia, Halomicronema, Kluyvera, Klebsiella, Kokuria, Komagataeibacter, Lactobacillus, Lactococcus, Luteomicrobium, Leuconostoc, Marivitia, Macrococcus, Mesorhizobium, Micrococcus, Microbacterium, Micrococcus, Novosphingobium, Providencia, Pediococcus, Pseudomonas, Rhodobacter, Rhodovulum, Ruminococcus, Sacrcina, Salinibacterim. Sarcandra, Serratia, Sphaerotilus, Sphingomonas, Sphingopyxis, Streptococcus, Streptomyces, Sulfuropirillum*, *Synechococcus*, *Tanticharoenia*, *Trochococcus, Weissella, Zymomonas*. **Yeasts**: *Bullera, Candida, Clavispora, Cryptococcus, Cystofilobasidium, Debaryomyces, Dekkera, Galactomyces, Hanseniaspora, Kazachstania, Kluyveromyces, Lipomyces, Meyerozyma, Pichia, Rhodotorula, Saccharomyces, Schwanniomyces. Torulaspora*, *Westerdykella, Wickerhamomyces, Zygosaccharomyces*. **Fungi**: *Aureobasidium*. *Aspergillus*, *Cladosporium*. *Penicillium*, *Rhizopus.*	Hñähñu, Ixcatec, Mazahua, Mixtec, Nahua, Ngiwa, Purhepecha, Triqui, Zapotec, Mestizo	[23,45,46,47,48,93,94,95,96,97]
**Tuba**	*Cocos nucifera* (sap)	**Bacteria**: *Bacillus*, *Cronobacter*, *Enterococcus*, *Erwinia, Fructobacillus*, *Gluconacetobacter*, *Klebsiella, Lactobacillus*, *Lactococcus*, *Leuconostoc*, *Micrococcus*, *Serratia, Sphingomonas*, *Vibrio*, *Zymomonas.* **Yeasts**: *Candida*, *Cryptococcus*, *Hanseniaspora, Saccharomyces.*	Mestizo	[96,98,99,100,101,102,103,104,105,106,107,108,109,110,111,112,113,114,115]
**Taberna**	*Acrocomia acuelata* (sap)	**Bacteria**: *Aerobacter*, *Acetobacter*, *Bacillus, Brevundimonas, Citrobacter, Enterobacter, Enterococcus, Fructobacillus, Gluconobacter, Klebsiella*, *Kluyvera*, *Lactobacillus*, *Lactococcus*, *Pantoea*, *Sphingomonas, Zymomonas. **Yeasts***: *Candida*, *Hanseniapora, Issatchenkia*, *Kazachstania*. *Meyerozyma*, *Pichia*. *Rhodotorula*, *Saccharomyces, Schizosaccharomyces*	Zapotec, Mestizo	[98,99,100]
**Tepache**	*Ananas comosus* (fruit)	***Bacteria***: *Acetobacter, Acinetobacter, Bacillus*, *Escherichia, Enterobacter, Enterococcus, Gluconobacter, Klebsiella, Lactobacillus, Lactococcus, Leuconostoc, Micrococcus, Pediococcusa, Weissella.* ***Yeasts**: Candida, Cryptococcus, Hanseniaspora, Meyerozyma, Pichia, Rhodotorula, Saccharomyces.***Fungi**: *Penicillium.*	Mestizo	[49,50,116,117,118]
**Colonche**	*Opuntia* spp. (fruits), *Pacchycerus, Stenocereus*	**Bacteria**: *Enterococcus*, *Lactobacillus*, *Leuconoctoc*, *Pediococcus, Weissella.***Yeasts**: *Candida*, *Hanseniaspora*, *Pichia, Saccharomyces.*	Chichimecan groups, Pame, Zapotec, Mestizo	[22,50,52,119]
**Mescal**	*Agave* spp.	**Bacteria**: *Acetobacter*, *Acinetobacter, Acetobacterium*, *Bacillus*, *Citrobacter*, Enterobacter, Erwinia, Chryseobacterium, *Gluconobacter*, *Kluyvera*, *Kokuria*, *Komagataeibacter*, *Lactobacillus*, *Lactococcus*, *Leuconostoc*, *Microbacterium*, *Providencia*, *Oenococcus*, *Pediococcus*, *Pseudomonas Serratia*, *Weissella*, *Zymomonas*. **Yeasts**: *Candida*, *Citeromyces*, *Clavispora*, *Cryptococcus*, *Debaryomyces*, *Dekkera, Diutinia, Hanseniaspora*, *Issatchenkia*, *Kazachstania, Kluyveromyces, Meyerozyma, Millerozyma,Naganishia, Ogataea, Pichia*, *Pseudozyma, Rhodosporidiobolus, Rhodotorula*, *Saccharomyces*, *Saturnispora*, *Schizosaccharomyces Sporidiobolus, Torulaspora*, *Trichosporon, Wickerhamomyces, Yamadazyma*, *Zygosaccharomyces.*	Cahiti, Guasave, Ixcatec, Pima, Tepehuan, Warohiro, Wixarika, Mestizo	[116,120,121,122,123,124,125,126]
**Chorote**	*Zea mayz (grains)* *Theobroma cacao* rosted beans),	*Fructobacillus*, *Lactobacillus*, *Leuconostoc*, *Gluconacetobacter*, *Sphingomonas*, *Vibrio*,	Maya, Mestizo	[127]
**Balché**	*Lonchocarpus* spp. *(bark and flowers)* and honeybee	**Yeasts**: *Saccharomyces.*	Lacandon, Maya	[84,128,129,130,131,132,133]
**Pox**	*Saccharum officinarum* and *Zea mays (stems)*	ND	Chol, Tzeltal, Tzotzil Mestizo	[53]
**Sambudia**	*Ananas comosus* (fruit)	ND	Mazahua, Mestizo	[134,135,136] [48,49,50,137,138,139,140,141]
**Sendechó**	*Zea mays (grains)* and *Capsicum* sp.	**Bacteria**: *Acetobacter, Bacillus, Enterococcus, Klebsiella, Kocuria, Lactobacillus, Leuconostoc, Micrococcus, Pediococcus, Pseudomionas, Staphylococcus, Zymomonas.***Yeasts**: *Candida, Clavispora, Cryptococcus, Galactomyces, Kluyveromyces, Rhodotorula, Saccharomyces*, *Torulaspora, Wickerhamomyces, Zygosaccharomyces.* **Fungi**: *Aureobasidium, Cladosporium, Epicoccum, Fusarium, Paecilomyces, Penicillum, Phima, Sclerotium, Verticillium.*	Mazahua, Hñähñu	

ND: No data recorded.

**Table 2:** Network values for plant substrates and microorganisms.

Network	Connectance	Links per Species	Niche Overlap	Mean of Shared Partners
Plant substrates	0.085	1.29	0.20	0.31
Microorganisms	0.23	1.75	0.99	7.21

## Data Availability

Data supporting the reported results are systematized in a database available by request to the first author.

## Supplementary Materials

The following are available online at www.mdpi.com/article/10.3390/foods10102390/s1, Table S1: Plants involved in the production of TMFB registered in the literature. Grey boxes represent the presence of the specie and white boxes the absence. ***Ato*** refers to ***atole agrio***, ***Bal*** to ***balché, Col*** to colonche, ***Mes*** to ***mescal***, ***Pox*** to ***pox, Poz*** to ***Pozol***, ***Sak*** to ***saká***, ***Sam*** to ***sambudia***, ***Sen*** to ***sendechó***, ***Tab*** to ***taberna***, ***Tub*** to ***tuba, Tep*** to ***tepache***, ***Tej*** to ***tejuino***, ***Tes*** to ***tesgüino***, ***Cho*** to ***chorote***, ***Pul*** to ***pulque***. Table S2. Microorganism’s genera previously registered in the literature. Grey boxes represent the presence of the genera and white boxes the absence. **Ato** refers to **atole agrio**, **Col** to **colonche**, **Mes** to **mescal**, **Poz** to **Pozol**, **Pul** to **pulque**, **Tej** to **tejuino**, **Tep** to **tepache**, **Tub** to **tuba**. The first three letters of the genera are displayed in the Figure 3.
