# Supplementary material for: Traditional Fermented Beverages of Mexico: A Biocultural Unseen Foodscape
Source: Foods. 2021 Oct 9;10(10):2390. doi: 10.3390/foods10102390 (PMC8535898; doi:10.3390/foods10102390)
Supplement: Supplementary file 1 [file foods-10-02390-s001.zip › foods-1394259-SI.pdf]

Table S1. Plants involved in the production of TMFB registered in the literature. Grey boxes represent the presence of the specie and white boxes the absence. ***Ato*** refers to ***atole agrio***, ***Bal*** to ***balché, Col*** to colonche, ***Mes*** to ***mescal***, ***Pox*** to ***pox, Poz*** to ***Pozol***, ***Sak*** to ***saká***, ***Sam*** to ***sambudia***, ***Sen*** to ***sendechó***, ***Tab*** to ***taberna***, ***Tub*** to ***tuba, Tep*** to ***tepache***, ***Tej*** to ***tejuino***, ***Tes*** to ***tesgüino***, ***Cho*** to ***chorote***, ***Pul*** to ***pulque***.

	Beverages															
Species	Ato	Bal	Col	Mes	Pox	Poz	Sak	Sam	Sen	Tab	Tub	Tep	Tej	Tes	Cho	Pul
Acrocomia acuelata										■						
Agave americana				■												■
Agave angustiarum				■												
Agave angustifolia				■												■
Agave applanata																■
Agave asperrima				■												■
Agave atrovirens				■												■
Agave bovicornuta				■												■
Agave cantala				■												
Agave cerulata				■												■
Agave chiapensis																■
Agave colorata																■
Agave convallis				■												
Agave cupreata				■												■
Agave deserti																■
Agave desmettiana				■												■
Agave duranguensis				■												
Agave fortiflora																■
Agave fourcroydes				■												■
Agave gentryi																■
Agave gigantensis				■												
Agave gudalajarana				■												
Agave hookeri																■
Agave horrida				■												■
Agave inaequidens																■
Agave jaiboli				■												■
Agave karwinskii				■												■
Agave kerchovei				■												■
Agave lechuguilla				■												■
Agave lopantha				■												
Agave lurida				■												
Agave lyobaa				■												
Agave macroacantha				■												■
Agave mapisaga				■												■
Agave margaritae				■												
Agave marmorata				■												■

Agave maximiliana
Agave megalodonta
Agave montana
Agave montium-sancticaroli
Agave multifilifera
Agave murpheyi
Agave nussaviorum
Agave oteroi
Agave palmeri
Agave parryi
Agave peacockii
Agave pintilla
Agave polianthiflora
Agave potatorum
Agave potatorum
Agave rhodacantha
Agave salmiana
Agave schotii
Agave seemanniana
Agave shawii
Agave shrevei
Agave sisalana
Agave sobria
Agave striata
Agave tequilana
Agave titanota
Agave triangularis
Agave valenciana
Agave victoriae-reginae
Agave vilmoriniana
Agave weberi
Agave wocomahi
Agave zebra
Ananas comosus
Anhalonium williamsi
Arachis sp.
Bromelia karatas
Bromus arozonicus
Byrsonima crassifolia
Cinnamomum verum
Citrus sinensis
Citurs sp.
Cocos nucifera

Crataegus mexicana
Cymbopogon sp.
Datura discolor
Dysphania ambrosioides
Echinocactus platyacanthus
Elaeis guineensis
Flreischmannia pycnocephalia
Hordeum vulgare
Ipomea batata
Ipomea batatas
Lippia Dulcis
Lonchocarpus longistylus
Lonchocarpus violasus
Machaerocereus gummosus
Malus domestica
Mentha spicata
Musa accuminata
Musa balbisiana
Musa sp.
Myristica fragrans
Myrtus communis
Opuntia amyclaea
Opuntia camuesa
Opuntia contrabrigiensis
Opuntia ficus-indica
Opuntia hiptiacantha
Opuntia leucotricha
Opuntia megacantha
Opuntia orbiculata
Opuntia robusta
Opuntia streptacantha
Orbignya cohune
Oryza sativa
Pacchycerus pringlei
Pacchycerus weberi
Phaseolus sp.
Pinus montezumae
Piper sp.
Psidum guajava
Raphia hookeri
Rubus sp.
Saccharum officinarum

Scheela sp.						■									
Sechium edule	■														
Sechium sp.						■									
Solanum	■														
Spondias mombin												■			
Stenocereus stellatus			■												
Stenocereus thurberi			■												
Syzygium aromaticum								■				■			
Tamarindus indica												■			
Telanthophora grandifolia	■														
Theobroma cacao						■									■
Zea mays	■				■	■	■	■	■				■	■	■
Chimaphila maculata														■	
Datura meteloides														■	
Hamaemelum nobile														■	
Cojoba arborea														■	
Stevia serrata														■	
Usnea dillenius														■	
Capsicum sp.								■							
Carnegia gigantea			■												
Escontria chiotilla			■												
Lonchocarpus punctatus		■													

Table S2. Microorganism's genera previously registered in the literature. Grey boxes represent the presence of the genera and white boxes the absence. ***Ato*** refers to ***atole agrio***, ***Col*** to colonche, ***Mes*** to ***mescal***, ***Poz*** to ***Pozol***, ***Pul*** to ***pulque***, ***Tej*** to ***tejuino***, ***Tep*** to ***tepache***, ***Tub*** to ***tuba.*** The first three letters of the genera are displayed in the Figure 3.

	**Beverages**							
Microorganisms	***Ato***	***Col***	***Mes***	***Poz***	***Pul***	***Tej***	***Tep***	***Tub***
Acetobacter			■		■			■
Acetobacterium					■			■
Achromobacter				■				
Acinetobacter					■			
Advenella			■					
Aerococcus	■	■						
Agrobacterium				■				
Alcaligenes				■				
Alternaria				■				
Aspergillus		■		■				

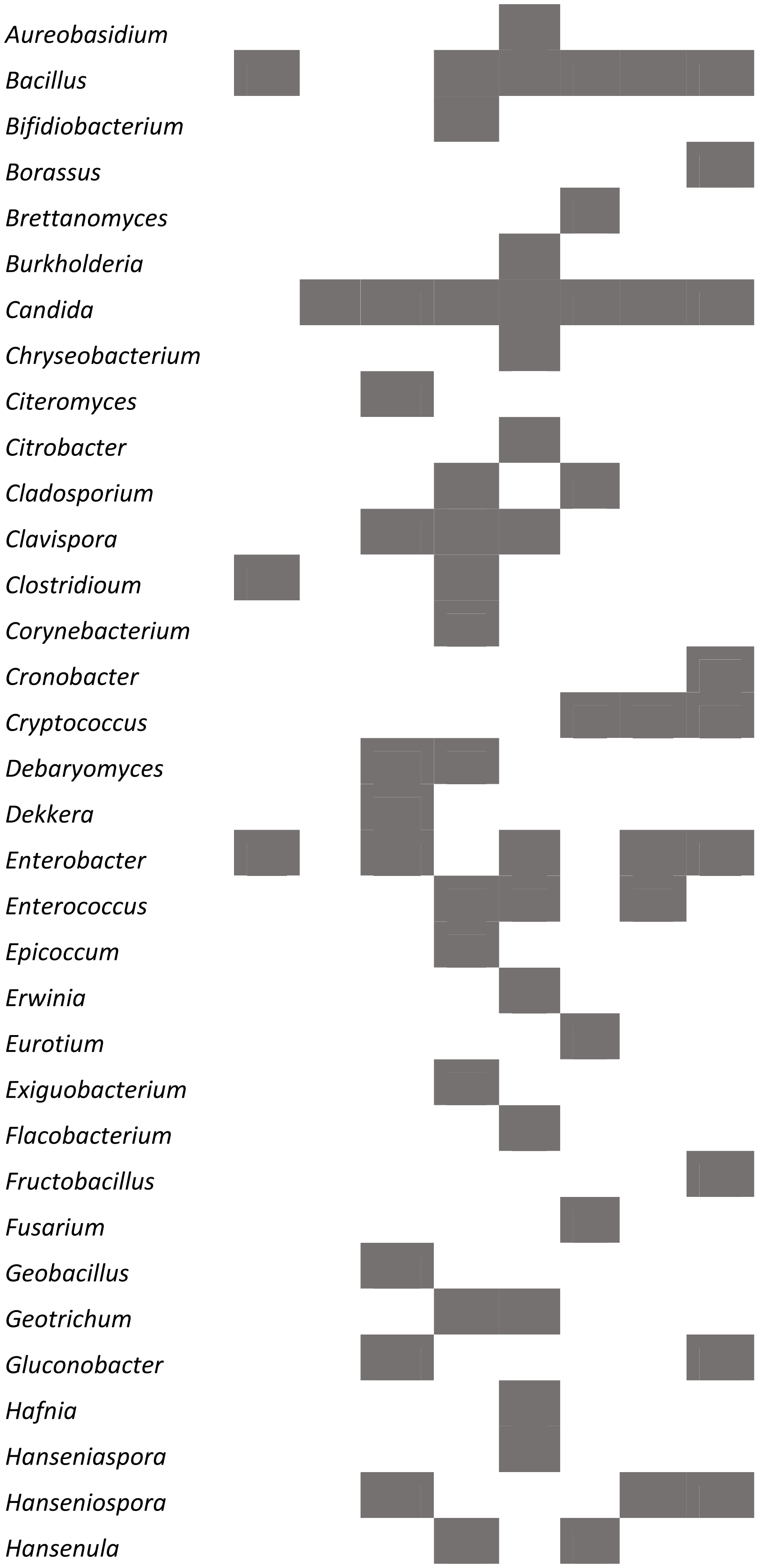
Aureobasidium
Bacillus
Bifidiobacterium
Borassus
Brettanomyces
Burkholderia
Candida
Chryseobacterium
Citeromyces
Citrobacter
Cladosporium
Clavispora
Clostridioum
Corynebacterium
Cronobacter
Cryptococcus
Debaryomyces
Dekkera
Enterobacter
Enterococcus
Epicoccum
Erwinia
Eurotium
Exiguobacterium
Flacobacterium
Fructobacillus
Fusarium
Geobacillus
Geotrichum
Gluconobacter
Hafnia
Hanseniaspora
Hanseniospora
Hansenula

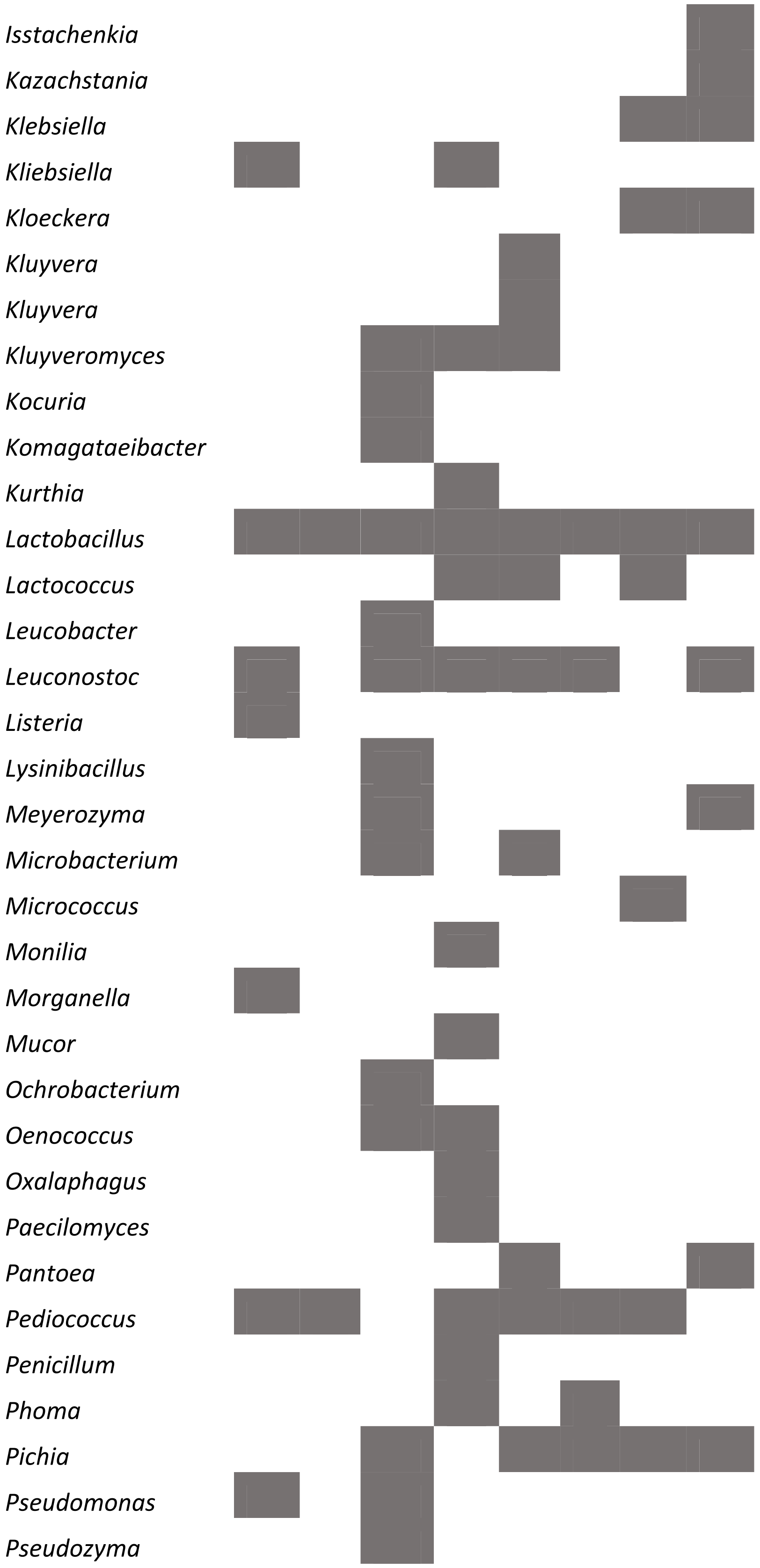
Isstachenkia
Kazachstania
Klebsiella
Kliebsiella
Kloeckera
Kluyvera
Kluyvera
Kluyveromyces
Kocuria
Komagataeibacter
Kurthia
Lactobacillus
Lactococcus
Leucobacter
Leuconostoc
Listeria
Lysinibacillus
Meyerozyma
Microbacterium
Micrococcus
Monilia
Morganella
Mucor
Ochrobacterium
Oenococcus
Oxalaphagus
Paecilomyces
Pantoea
Pediococcus
Penicillum
Phoma
Pichia
Pseudomonas
Pseudozyma

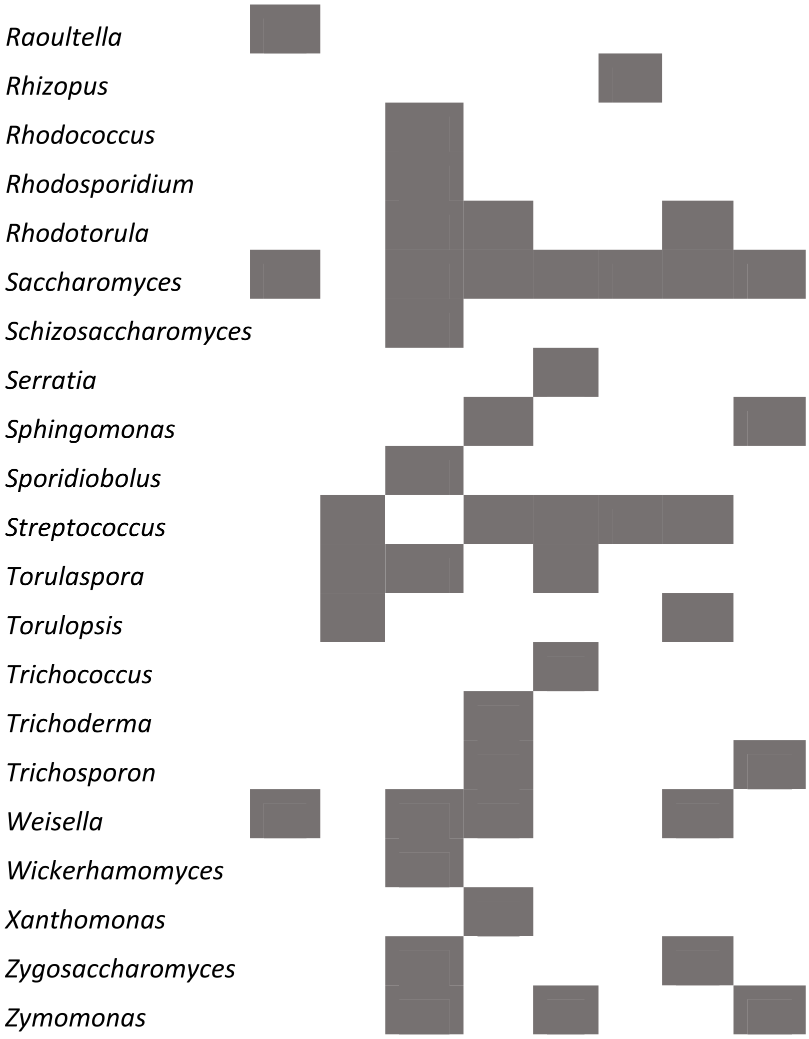
Raoultella
Rhizopus
Rhodococcus
Rhodosporidium
Rhodotorula
Saccharomyces
Schizosaccharomyces
Serratia
Sphingomonas
Sporidiobolus
Streptococcus
Torulaspora
Torulopsis
Trichococcus
Trichoderma
Trichosporon
Weisella
Wickerhamomyces
Xanthomonas
Zygosaccharomyces
Zymomonas
